# Social Influence and Different Types of Red-Light Behaviors among Cyclists

**DOI:** 10.3389/fpsyg.2016.01834

**Published:** 2016-11-22

**Authors:** Federico Fraboni, Víctor Marín Puchades, Marco De Angelis, Gabriele Prati, Luca Pietrantoni

**Affiliations:** Department of Psychology, University of BolognaBologna Italy

**Keywords:** cycling behaviors, social validation, social influence, group pressure, red-light violations, road safety

## Abstract

Accident analysis and studies on traffic revealed that cyclists’ violation of red-light regulation is one typical infringement committed by cyclists. Furthermore, an association between cyclists’ crash involvement and red-light violations has been found across different countries. The literature on cyclists’ psychosocial determinants of red-light violation is still scarce. The present study, based on the classification of cyclists’ red-light behavior in risk-taking (ignoring the red-light and traveling through the junction without stopping), opportunistic (waiting at red-lights but being too impatient to wait for green signal and subsequently crossing the junction), and law-obeying (stopping to obey the red-light), adopted an eye-observational methodology to investigate differences in cyclists’ crossing behavior at intersections, in relation to traffic light violations and the presence of other cyclists. Based on the social influence explanatory framework, which states that people tend to behave differently in a given situation taking into consideration similar people’s behaviors, and that the effect of social influence is related to the group size, we hypothesized that the number of cyclists at the intersection will have an influence on the cyclists’ behavior. Furthermore, cyclists will be more likely to violate in an opportunistic way when other cyclists are already committing a violation. Two researchers at a time registered unobtrusively at four different intersections during morning and late afternoon peak hour traffic, 1381 cyclists approaching the traffic light during the red phase. The 62.9% violated the traffic control. Results showed that a higher number of cyclists waiting at the intersection is associated with fewer risk-taking violations. Nevertheless, the percentage of opportunistic violation remained high. For the condition of no cyclist present, risk-taking behaviors were significantly higher, whereas, they were significantly lower for conditions of two to four and five or more cyclists present. The percentage of cyclists committing a red-light violation without following any other was higher for those committing a risk-taking violation, whereas those following tended to commit opportunistic violations more often.

## Introduction

Using bicycle as a transport mode is healthy, cheap, and environmentally friendly. In Europe, 8% of people choose bicycles as the most common mode of daily transport (European Commission, 2014). Nevertheless, cyclists still represent one of the road user categories with the highest risk of injuries and fatalities (European Road Safety Observatory, 2015). From 2004 to 2013, cyclists’ fatalities decreased by 32%, but from 2010 this tendency has stagnated, with less than a 1% year-to-year reduction. Furthermore, 31% of the fatalities happen at junctions (European Road Safety Observatory, 2015). Risks for non-fatal accidents are higher for cyclists than for car drivers, as shown by De Hartog et al. (2010).

Similar to European data, in Italy, 6% of the population indicates the bicycle as the most common mode of transport (European Commission, 2014). In 2014, there were 18.055 bicycle accidents and 273 fatalities recorded (Automobile Club d’Italia - ISTAT, 2014). The mortality index (deaths every 100 accidents) for cyclists is 1.42, which is more than double compared to car users (Italian National Institute of Statistics [ISTAT], 2015). Moreover, 38% of these accidents take place at junctions (European Road Safety Observatory, 2015).

Accident analysis reveals that violation of traffic rules plays a key role in fatal crashes involving cyclists. Red-light violation is one typical violation behavior among cyclists (Wu et al., 2012; Pai and Jou, 2014). Specifically, the rate of red-light violations among cyclists has been measured in different countries and cultures, varying from the 6.9% rate of red violations in Melbourne (Johnson et al., 2011) to the 87.5% in Dublin (Lawson et al., 2013). Schramm et al. (2010), using police-reported bicycle crashes in Queensland, Australia, found that the most frequently recorded cyclists’ traffic violation was “disobey traffic light” (6.4%). In addition, Thom and Clayton (1992) found that even if disobeying stop sign or red-light was recorded only in the 2.4% of the observation, it accounted for the 11.1% of the accidents. Several studies have shown an association between cyclist crash involvement and red-light violations (Retting et al., 1999; Johnson et al., 2013). Cyclists’ violations at intersections (e.g., bicyclists ride out and ride through at signalized intersections during the red phase) are estimated to account for the 8.8% of total bicyclists’ crashes among North Carolina municipalities (University of North Carolina - Highway Safety Research Center, 2014).

Several authors have delved into psychological and social determinants of red-light violations of different road users. Understanding the factors associated with each type of violation (e.g., social influence) can help craft better policies and develop appropriate interventions to prompt cyclists to respect the red-light signal and possibly, reduce the number of traffic accidents due to them.

The literature focusing on cyclists is still scarce, while studies on pedestrian crossing behavior are more frequent. In his study, Rosenbloom (2009) observed pedestrians’ red-light crossing and showed that, the presence of other pedestrians waiting at the crosswalk upon a pedestrian’s arrival, as well as the arrival of other pedestrians to the crosswalk, decreased the likelihood of crossing on a red-light. Also van der Meel (2013) suggested that pedestrians tend to wait for the red-light more often when there are other pedestrians waiting. Rosenbloom (2009) argued that people would feel higher commitment in respecting social norms when they are grouped, thus complying more with the law, whereas, when alone, people are less concerned with the social criticism and will violate the law more easily. As it is true for socially accepted behaviors, the presence of similar others has been found to have an influence on deviations from the norm. For example, Mulders and Oude Egberink (1984) reported that when other people ran the red-light, the pedestrians approaching the traffic light were more likely to violate it. Other studies on young pedestrians found subjective norms (perceived social pressure) to be a significant predictor of potentially hazardous road crossing behaviors (Evans and Norman, 1998, 2003).

For what concerns cyclists, Wu et al. (2012) found that the probability of a rider running a red-light was higher when she or he was alone, when there were fewer riders waiting, and when there were riders already crossing on red. Johnson et al. (2011) found that the presence of other road users, cyclists and drivers, traveling in the same direction had a deterrent effect on cyclists’ red-light infringements. Similarly, in an older study (Bureau Goudappel Coffeng, 1985), the presence of other cyclists was associated with a decreased probability of infringement by the observed cyclists.

This phenomenon can be explained according to the social validation principle of social influence, which states that people tend to consider the appropriateness and correctness of their behaviors in a given situation taking into consideration similar people’s behaviors (Cialdini and Griskevicius, 2010). This formulation derives from classic literature findings in Social Psychology, stating that individuals decide on appropriate behaviors for themselves in a given situation by searching for information as to how similar others have behaved or are behaving in that situation (Asch, 1956; Darley and Latane, 1970). In the case of cyclists at junctions, for example, they could be prompted to stop at the red-light because they can see other cyclists waiting at the traffic light, or they could skip the red-light following another cyclist that violated the traffic signal right in front of them (i.e., following behavior).

Social influence has been shown to be dependent on group size. The findings from the literature regarding the effect of the group size on red-light behaviors are unclear: whereas some authors (Bureau Goudappel Coffeng, 1985; Wu et al., 2012; Dommes et al., 2015) found group pressure on red-light running behaviors to increase in a larger group, van der Meel (2013) did not find any relation between group size and red-light violations.

One reason for these inconsistencies could have to do with the fact that red-light violations may have different characteristics and cannot be included in one category. Pai and Jou (2014) classified bicyclists red-light crossing behaviors into three types: the (1) risk-taking behavior, that is, ignoring the red-light and traveling through the junction without stopping (but may slow down); the (2) opportunistic behavior, that is, waiting at red-lights but being too impatient to wait for red-lights to turn green and subsequently crossing the junction by seeking gaps among conflicting traffic streams; and the (3) law-obeying behavior, that is, stopping to obey the red-light. The main difference between risk-taking and opportunistic behaviors is the timing of the violation. In risk-taking behavior, the cyclist crosses the street immediately without stopping at the junction. In opportunistic behavior, the cyclist at first stops at the intersection and subsequently crosses the street. Distinguishing the two different types of red-light violations (i.e., opportunistic and risk-taking) is of utmost importance in order to understand the different levels of risk entailed by different behaviors. Since the opportunistic category involves a stop at the intersection and a violation of the red-light after an evaluation of the situation and eventually the identification of relatively “safe gaps” in the traffic flow, it is considered less dangerous compared to risk-taking behavior (Johnson et al., 2008). Indeed, risk-taking behavior refers to crossing the intersection without stopping or slowing down and, therefore, leaving less time to identify risks and take necessary maneuvers to avoid accidents.

Intentions or motivations behind each type of violation might differ. For instance, Pai and Jou (2014) stated that opportunistic violations take place when cyclists become impatient while waiting at the traffic light and try to find traffic gaps to commit the violations. In our opinion, social influence could also differently predict the three types of red-light behaviors.

To further understand this phenomenon, we want to explore whether group size is associated with higher or lower incidences of different types of red-light behaviors. As suggested by van der Meel (2013), further research on different group size is needed to confirm the effect larger groups have on cyclists’ red-light running. Asch (1955) and later researchers found that conformity increases as the number of people in the group increases, but once the group reaches four or five other people conformity does not increase much (Gerard et al., 1968; Campbell and Fairey, 1989; Bond, 2005). Considering this, we will address the recommendation by van der Meel (2013), investigating the effect of group size on different type of cyclists’ red-light behaviors. Cyclists tend to be influenced by two important group norms: injunctive norms involve perceptions of which (traffic) behaviors are typically approved or disapproved and they assist an individual in determining what is acceptable and unacceptable; descriptive norms involve perceptions of which (traffic) behaviors are typically performed and they normally refer to the perception of others’ behavior. Cialdini (2009) described the use of descriptive social information as the decision-making heuristic of social proof. Using this information as a heuristic cue for behavior, cyclists who see other cyclists stopping at the intersection are supposed to be less likely to commit risk-taking violations. As a consequence, we hypothesize that a higher number of cyclists waiting at the intersection will be associated with fewer risk-taking violations compared to the two other types of behavior (Hypothesis 1).

As the presence of other people will prompt cyclists to stop in the first place, we hypothesize that there will be other cyclists following (i.e., following behavior) when other cyclists start committing violations, therefore committing more opportunistic violations (i.e., after having stopped). Even if only one cyclist within the group of cyclists waiting at the crossroad commits a red-light violation, this could be sufficient to elicit the same behavior in other cyclists. Conformity studies have systematically shown that when the majority is not unanimous, its influence is reduced (Asch, 1955, 1956). Moreover, a cyclist that commits a red-light violation could also be considered a social supporter who sided with minority members (e.g., those cyclists who do not obey to traffic rules). Previous studies revealed that social support for non-conformity reduced conformity (see Allen, 1975, for a review).

Thus, we hypothesize that following behavior will be significantly associated with opportunistic violations rather than with risk-taking violations (Hypothesis 2). In other words, we expect that the frequency of cyclists committing a red-light violation following another cyclist will be higher for those committing an opportunistic violation than for those committing a risk-taking violation.

## Materials and Methods

### Procedure

In this cross-sectional study, we adopted an eye-observational methodology to investigate differences in cyclists’ behavior at intersections, in relation to traffic light violations and the presence of other cyclists.

**FIGURE 1:**
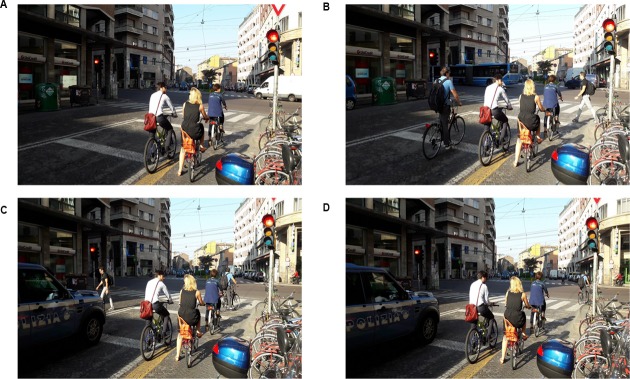
**A cyclist violating the red-light in an observation site.** Photo **(A)** top left; photo **(B)** top right; photo **(C)** bottom left; photo **(D)** bottom right.

Our observations took place in the urban area of the city of Bologna, Italy. We selected the intersections basing on two main criteria: (a) high scores of bicycle traffic flow on cycling facilities; (b) most common type of cycling infrastructure in the Municipality of Bologna. According to previous research (Du et al., 2013; Yang et al., 2014), we selected the sites satisfying the following requirements: the presence of pedestrian crossing and bicycle lanes; enough distance between observation sites so the same cyclists were unlikely to be observed twice; and less likelihood of interfering with observed behaviors. Furthermore, regarding the structural aspects of the crossing at the selected intersections, it is important to mention that they allow for a red-light violation even when there are about three or more cyclists waiting at the traffic light. The selected four sites did not have any kind of physical barriers between the cycle lane and the main road, enabling an increased freedom of movement for cyclists. **Figure 1** displays one typical red-light violation committed by a cyclist in one of the observational sites. In photo (A) a group of cyclists stops at the intersection and waits for the green-light, in photo (B) a cyclist approaches the intersection, while in photos (C,D) the cyclist disregards the traffic light and decides to cross the road.

Five observers, who had previous experiences in observational studies, were selected for the present study. Before the actual observations, the observers were trained to maximize the inter-rater agreement in coding the different behaviors, and to guarantee data quality control. After the training phase, two randomly selected observers were asked to go in one of the four observation sites and to assess the same cyclists at the same time, during a 1-hour session. Inter-rater agreement was excellent for gender (Cohen’s Kappa = 1.000), presence of other cyclists (Cohen’s Kappa = 1.000) and following behavior (Cohen’s Kappa = 1.000). The agreement was very good for red-light behavior (Cohen’s Kappa = 0.951) and for age (Cohen’s Kappa = 0.848).

Two observers at time were randomly assigned to different sites and peak times, changing both the observational periods and sites every time. To obtain a greater amount of information about red-light behaviors, the two observers were instructed to observe the behaviors of different cyclists at the same intersection at the same time. Thus, the setting was the same for both observers but the two observers coded the behavior of different cyclists. **Table 1** summarizes the work observation plan for the four intersections selected.

**Table 1:** Observation plan.

Site	Time of the day	Hours of observations	Number of cyclists (%)
Site 1	Morning (2) and Evening (1)	4.5 h	210 (15.2%)
Site 2	Morning (1) and Evening (2)	4.5 h	365 (26.4%)
Site 3	Morning (1) and Evening (2)	4.5 h	331 (24.0%)
Site 4	Morning (2) and Evening (2)	6 h	475 (34.4%)

*Morning comprises from 8:30 to 9:00 a.m.; Evening comprises from 17:00 to 18:30 p.m. The number between parenthesis after Morning and Evening corresponds to the number of observations per each time of the day.*

The observational survey was made between the 5th of April 2016 and the 29th of April 2016, during peak hours and weekdays. Considering the daily variance of traffic characteristics, we randomly selected the days, setting 1.30 h intervals for each observation made. The time of day included two peak times (from 8.00 to 9.30 a.m., and from 5.30 to 7.00 p.m.), during which traffic flow was previously investigated (Rupi, 2015). Furthermore, the observations generally tend to replicate the cyclists’ commuters flow, considering cyclists’ commuters are both workers and university students. Consistent with this, we chose April because the cyclists’ flow appears to be higher during spring (Thomas et al., 2013).

Before starting with the observation, the two researchers had to specify the site and infrastructure characteristics. Only the cyclists who approached the intersection during the red-light phase were coded, since we were interested in observing the red-light behaviors.

### Measures

We collected the data through a Web App built via Qualtrics software running through a smartphone. We tested the instrument at an intersection that fulfilled the inclusion criteria. The App was configured in a way to collect the data of one cyclist and immediately refresh the page for a new observation. The App was designed in a way that one survey contained the data regarding one cyclist. Each observer had to assess the following variables.

#### Observation Site

The observers had to select the respective site in which the observation took place (1 = San Donato; 2 = Bassi/Indipendenza/Rizzoli; 3 = Riva di Reno/Via Marconi; 4 = Sabotino).

#### Red-Light Behavior

In order to get a deeper insight on how the cyclists behave when approaching red-lights, we adopted the same classification used in the study by Pai and Jou (2014), addressing three types of behavior, each entailing a different level of risk: (1) risk-taking; (2) opportunistic; and (3) law-obeying behavior. Observers had to assess the cyclists’ behavior using three options (0 = The cyclist complied with the red-light signal; 1 = The cyclist initially stopped at red-light but then crossed the intersection before the green light; 2 = The cyclist ran straight through the red-light).

#### Presence of Other Cyclists

As previously done differently in other studies (Wu et al., 2012), we were interested in assessing if the presence of other cyclists waiting at the intersection could have an effect on cyclists’ red-light compliance. The variable was assessed through a multiple-choice question: “how many other cyclists were already waiting at the red-light when the cyclists approached?” (0 = no cyclists; 1 = one cyclist; 2 = from two to four cyclists; 3 = five or more cyclists).

#### Following Behavior

Observers were asked to assess if the cyclist was committing a violation right after another cyclist (0 = No; 1 = Yes).

*Age and gender* were also assessed based on an estimation from each observer (0 – 30 years old; 31–50 years old; 50+ years old).

### Participants

We observed 1381 cyclists approaching the traffic light during the red phase, 704 (51.0%) were male and 647 (48.7%) were female, four observations (0.3%) count as missing. Of the total sample, 504 participants were 30 years old or younger (36.5%), 561 were within 31 and 50 years old (40.6%), and 315 were as older than 50 (22.5%).

## Results

We used SPSS version 23 to carry out all the statistical analyses. Regarding the type of red-light behavior, 512 (37.2%) cyclists did comply with the red-light and waited until it switched to green, 409 (29.6%) committed an opportunistic violation, and 460 (33.3%) committed a risk-taking violation. **Table 2** displays the frequency of the number of cyclists present at the intersection, whereas **Table 3** shows the frequencies of the three types of red-light behaviors by the number of cyclists at the intersection.

**Table 2:** Frequencies and percentages of following behavior and cyclists present.

Variable	Frequency (percentage)
Cyclists present^1^	*N* = 1381
0	528 (38.2%)
1	333 (24.1%)
2–4	430 (31.1%)
5+	85 (6.2%)

*^1^Missing values are 5 (0.4%).*

**Table 3:** Red-light behavior frequencies by the presence of cyclist.

	Red-light behavior					
	**Law-obeying**		**Opportunistic**		**Risk-taking**	
	***n***	**%**	***n***	**%**	***n***	**%**

Cyclist present						
0	160_a_	30.3%	121_a_	22.9%	247_b_	46.8%
1	116_a_	34.8%	110_a_	33.0%	107_a_	32.1%
2–4	182_a_	42.3%	149_a_	34.7%	99_b_	23.0%
5 or more	53_a_	62.4%	27_a_	31.8%	5_b_	5.9%

*Different subscripts within the same row correspond to proportions that differ significantly from each other (p < 0.05).*

To analyze the number of cyclists undertaking “following behavior” we only considered the subsample corresponding to the cyclists that skipped the red-light (*n* = 869). The rationale behind this is that it was a requirement for a given observation to be registered as following/not following. Among these, 235 (27%) followed another cyclist while committing the violation, whereas 623 (71.7%) did not. Data were missing for the rest of the cases (i.e., 11 observations).

In Hypothesis 1, we stated an association between the number of cyclists present at the intersection and the risk-taking violations insofar as we expected higher presence of cyclists to be related to fewer risk-taking violations. After carrying out chi-square test, we found a significant association between cyclists present and the type of risk-taking behavior χ^2^ (6) = 99.73, *p* < 0.001. **Table 3** shows the frequencies of each type of behavior by the number of cyclists present. Bonferroni comparisons showed that for the condition of no cyclist present, risk-taking behaviors were significantly higher (*p* < 0.05), whereas, they were significantly lower for conditions of two to four and five or more cyclists present (*p* < 0.05).

To test Hypotheses 2, we used a subset of the sample that had committed a violation (*n* = 869). The rationale for this choice was that this hypothesis involved either following or not following behavior, and therefore, it implied the cyclist to have committed a violation in the first place.

In Hypothesis 2, we suggested an association between following behavior and opportunistic type of violation, to the extent that following behaviors will take place in conjunction with opportunistic violations rather than with risk-taking ones. To this end, we performed a chi-square test on the cases involving red-light violation. Chi-square test showed that following behavior was significantly associated with opportunistic violations rather than with risk-taking ones χ^2^ (1) = 24.29, *p* < 0.001. **Table 4** displays the frequencies and percentages of cyclists per following behavior and type of violation. Bonferroni comparisons showed that, whereas the percentage of cyclists committing a red-light violation without following any other was higher for those committing a risk-taking violation (*p* < 0.05), those following (i.e., following behavior) tended to commit opportunistic violations more often (*p* < 0.05).

**Table 4:** Following behavior frequencies by the red-light behavior.

	Red-light behavior			
	**Opportunistic**		**Risk-taking**	
	***n***	**%**	***n***	**%**

**Following behavior**				
No	262_a_	42.1	361_b_	57.9
Yes	143_a_	60.9	92_b_	39.1

*n = 869. Different subscripts within the same row correspond to proportions that differ significantly from each other (p < 0.05).*

## Discussion

The main goal of this study was to bring further insight into the relationship between social influence and different cyclists’ red-light behaviors. In Hypothesis 1, we proposed that the higher the number of cyclists waiting at intersection, the lower the prevalence of risk-taking violations would be. This hypothesis was confirmed, thus, implying that a lower presence of cyclists waiting at intersection is associated with riskier red-light behaviors (i.e., risk-taking). We also found that the percentage of cyclists that obey the red-light signal increases with group size.

Our findings are supported by previous literature on cyclists’ red-light behavior (Bureau Goudappel Coffeng, 1985; Rosenbloom, 2009; Johnson et al., 2011; Wu et al., 2012; van der Meel, 2013), although they extend it by linking the size of the group and the type of red-light behavior (i.e., also the type of violation). Thus, we found that a higher number of cyclists reduces the prevalence of risk-taking violations, but it is not so for the opportunistic ones. In contrast, previous research had just found that the presence of cyclists is associated with fewer red-light violations (Bureau Goudappel Coffeng, 1985; Johnson et al., 2011; Wu et al., 2012; van der Meel, 2013), without specifying the type of behavior. The influence of other cyclists stopped at intersection leads other cyclists to conform to them because they see those people as a valuable source of information in guiding their behavior. People generally believe that similar others’ interpretations of an ambiguous set of circumstances are accurate and will help them choose an appropriate course of action.

We found that the effect varies with group size, and thus group pressure influences cyclists crossing behavior as previously found by the Bureau Goudappel Coffeng (1985). The present study extends this previous research addressing which type of behavior is fostered by the increasing number of cyclists waiting at intersection, giving us a deeper insight into the normative pressure it exerts, explaining the effect of social influence on crossing behavior.

In Hypothesis 2, we foresaw that there would be an association between following behaviors and opportunistic violations. This hypothesis was confirmed, thus, entailing that cyclists tend to violate following others when they have previously stopped and see another cyclist committing a violation. In such case, we can assume the role played by social support for non-conformity with the norm (i.e., not skipping the red-light). This way, cyclists, probably after assessing the potential risk of crossing a red traffic light, follow other cyclists that started crossing in the first place. It remains undetermined whether the decision of crossing is due to a lowered risk perception after seeing another person doing so, or if it is entirely due to social support to non-conformity (i.e., the behavior turns more acceptable after being undertaken by another cyclist).

The present study contributes to extend Wu et al.’s (2012) results, confirming that the number of cyclists present at the intersection, and how they act, has an influence on cyclists’ red-light behavior. The added value of our findings is that they contribute to explain how cyclists behave differently when crossing at signalized intersections, and whether seeing another cyclist violating could differently predict the two types of red-light violations. This study confirms that there is an effect of social influence in road crossing situations, regardless of the safety or the risk of the behavior. On the one hand, the decision-making heuristic of social proof (Cialdini, 2009) explains why cyclists behave differently when they are in groups than when alone: cyclists that approach a red-light when other cyclists are already there, will be more prone to stop because they can actually see similar others adopting a law-obeying behavior, and, thus, will use this information as a heuristic cue for behavior. On the other hand, opportunistic behavior could be more likely if one or more cyclists of the group decide not to wait until the green-light. This is because social support for non-conformity reduces conformity and, this way, minority members could influence majority members (Allen, 1975). Minority members can become a “model of dissidence” and may induce people to break “the social contract, the rules of the social game according to which individuals must conform to the majority” (Moscovici and Mugny, 1983, p. 53).

Social influence has been reported to have an influence on crossing behavior also in other studies (e.g., Yagil, 2000; Rosenbloom, 2009). To further elaborate our results, we can discuss them using Social Control Theory (Hirschi, 1969), according to which, the mechanism behind the cyclists’ law-obeying behavior is the motivation to be rewarded, or not to be punished, just for conforming. In this particular case, the main concern of the cyclists could be to avoid social criticism. As Hirschi and Gottfredson (1994) argued, in some cases, the sanctions of society (i.e., social criticism) are grater deterrents for normative people than are formal sanctions, especially for categories such as cyclists, which are not so strictly targeted by law enforcers. Furthermore, cyclists that are alone when approaching the intersection during the red-light phase, are less concerned with social criticism and, therefore, more likely to show risky behavior.

There could be other variables influencing cyclists’ red-light behavior, which the present study does not take into account. For example, the oncoming traffic volume or the traffic speed, neither of them considered in the present study, may influence the cyclists’ red-light behavior (Harrell, 1991; Yagil, 2000; Yang et al., 2006; van der Meel, 2013). Furthermore, to better understand the effect of social pressure, the present study should be complemented by data on peoples’ attitudes and beliefs concerning traffic light violations and, more generally, the obedience of the law. The sample of the current study does not include children aged less than 15 years old because of the very low number of observations for this age category. Including them could have shown different trends among the considered age groups. For example, another study (Ben-Moshe, 2003, unpublished) that examined the road crossing decisions of young children and adolescents (6, 9, and 13-year-old boys and girls) revealed that participants standing with their peer group on a crosswalk show much laxer attitude toward risk-taking when crossing the street, than the same participants standing alone. Thus, the findings of the study highlight that the mechanism of social validation (Cialdini and Griskevicius, 2010) works differently when teenagers are involved. Other studies (Christensen and Morrongiello, 1997; Miller and Byrnes, 1997) confirmed those findings, showing the adolescent’s tendency to take more risks in the presence of their peer group.

### Limitations

Care should be taken when interpreting the findings of the present study because of several limitations. First, this study is limited by a lack of generalizability to other settings (e.g., different regions as well as different countries) or to other conditions (e.g., different weather conditions or during off-peak hours). For instance, the frequency of red-light violations may be different from that of other settings. Second, although we have selected different settings and coded their similarities and differences, we cannot rule out the possibility that potential bias due to confounding variables (e.g., road infrastructure characteristics) exists. Third, in the present study we have employed a field observation approach that does not allow for an investigation of cyclists’ perception, expectations, and attitudes underlying their decisions and behaviors at the traffic light. Those data could have only been gathered by interviewing the people who committed the violations or conducting surveys in the area where the observations took place. Future studies using survey methods are needed to gain a more comprehensive understanding of cyclists’ behavior at traffic light. Fourth, since each observation in the present study accounted for different cyclists, and the same cyclist has not been observed twice in different conditions, there is no possibility to actually tell if there is a tendency for each single person to perform differently in the presence of similar others than when alone.

### Practical Implications

The fact that the number of cyclists waiting is associated with fewer risky behaviors and violations may have some practical implications. For instance, this effect could be taken into account in urban planning and policy-making. This way, decision makers could consider clustering the main cycling lanes into the main routes and widening them so that they can hold a higher flow of cyclists. This way, there would be a higher probability of finding others at intersections. Nevertheless, research on this solution would be needed since other factors might be affecting cyclists’ behavior (e.g., eventual use of sidewalks). Grouping and platooning cyclists through traffic control systems (e.g., green waves) could be a further solution.

Another potential solution may be the use of a countdown signals. Countdown devices have been increasingly applied and their influence on driver reaction and behavior at signalized intersections has been investigated on drivers and pedestrians but little on cyclists. The main purpose of countdown devices is to draw the road user’s attention to the quantity of time still available for a given light phase. This information allows them to better prepare for the starting and stopping phases. There is evidence showing that a red and green light countdown display might increase the ratio of pedestrians that finish crossing before the end of the green light (Ma et al., 2015) and reduce the prevalence of illegal crossing among pedestrians (Lipovac et al., 2013). Moreover, countdown displays for both green and red-light phases reduce the number of violations committed by motor vehicle drivers during the beginning of the red phase (Limanond et al., 2010). Nevertheless, for it to increase traffic light compliance among cyclists, conditions such as waiting time and time of the day should be taken into account. For instance, Pai and Jou (2014) found that, during off-peak hours, countdown signals with duration of 30 s increase bicyclists’ opportunistic behaviors.

Institutions should consider implementing campaigns to increase peoples’ negative injunctive norm on red-light skipping. This means to better explain through signs and advertisements that red-light violation is a behavior not approved at a community level. In fact, Lawrence (2015) found that injunctive norm messages could be effective in reducing phone-related distracted driving, but only when they draw people’s attention to social disapproval of that behavior.

Hirschi (2004) assumes that strengthening the ties to conventional social institutions might increase the commitment of individuals to normative behavior. Authorities might be willing to apply this principle by implementing public educational programs to increase self-control and, hence, normative and safer behavior.

## Conclusion

The present study examined the relationship between different types of cyclists’ red-light behavior and the presence of similar others at intersection. Results showed a high rate of red-light violation by cyclists. Both hypotheses have been confirmed, revealing that the presence of other cyclists stopped at intersection prompts cyclists to stop and that when cyclists are alone at the traffic light, they are more likely to skip the red-light in a risk-taking manner. Furthermore, results highlighted that cyclists tend to violate following others when they have previously stopped and see another cyclist committing a violation. These findings go beyond previous studies, giving more insight into the effect of group size on different types of red-light behavior. This study provides some practical suggestions to policy makers and traffic planners to help them design effective interventions and education programs to reduce red-light violations committed by cyclists and, consequently, possible accidents and injuries.

## Author Contributions

All authors listed contributed equally to the development of the study.

## Conflict of Interest Statement

The authors declare that the research was conducted in the absence of any commercial or financial relationships that could be construed as a potential conflict of interest.
